# The Vaccination Papers Ought to Be Welcomed

**Published:** 1898-06

**Authors:** W. B. Clarke

**Affiliations:** Indianapolis, Indiana


### THE VACCINATION PAPERS OUGHT TO BE WELCOMED.

INDIANAPOLIS, INDIANA.

EDITOR OF THE HOMŒOPATHIC PHYSICIAN:—Noting your request for opinions regarding the continuance of articles on vaccination, I wish to record my approval of them, and hope the entire subject will be thoroughly ventilated. Surely the vaccinationists, who are so confident of the efficacy of their pet, ought to welcome the fullest investigation of its merits, that it will shine all the brighter if it emerges unsullied. It is, as the motto of your journal puts it, "He is a freeman whom truth makes free, and all are slaves beside." "Seek the truth, come whence it may, cost what it will."

W. B. CLARKE, M. D.

